# When Dialysis “Becomes Life”: Pediatric Caregivers' Lived Experiences Obtained From Patient-Reported Outcomes Measures

**DOI:** 10.3389/fped.2022.864134

**Published:** 2022-05-23

**Authors:** Daniella Levy Erez, Melissa R. Meyers, Swathi Raman, Melissa Thomas, Susan Furth, Christopher B. Forrest, Michelle Denburg

**Affiliations:** ^1^Department of Pediatrics, The Children's Hospital of Philadelphia, Philadelphia, PA, United States; ^2^Perelman School of Medicine, University of Pennsylvania, Philadelphia, PA, United States; ^3^Schneider Children's Medical Center, Petah Tikva, Israel; ^4^Division of Nephrology, Children's National Medical Center, Washington, DC, United States; ^5^George Washington University School of Medicine, Washington, DC, United States

**Keywords:** chronic dialysis, child, caregiver, patient-reported outcomes measurement information system (PROMIS), quality of life, patient reported clinical outcomes

## Abstract

**Introduction:**

Qualitative research reveals significant caregiver impact resulting from managing children requiring chronic dialysis but offers few quantitative measures of their lived experiences.

**Materials and Methods:**

This cross-sectional study included 25 caregivers of children on chronic peritoneal dialysis (PD) and hemodialysis (HD) enrolled from 2018 to 2019 at a large pediatric dialysis program in the U.S.

Patient Reported Outcomes Measures Information System (PROMIS) measures and free text commentary were collected and analyzed to evaluate the self-reported impact and wellbeing of these caregivers.

**Results:**

Among all dialysis modalities, caregivers' positive affect (43.4 ± 10) and general life satisfaction (45.1 ± 11.5) were significantly lower than the general adult population. Compared with HD caregivers, PD caregivers demonstrated significantly more fatigue and sleep disturbance and less positive affect and life satisfaction. Amongst HD caregivers, sleep disturbance, positive affect, and meaning/purpose differed significantly from the general population. Analyses of text commentary revealed that caregivers also expressed the feelings of loss, importance of knowing the impact of dialysis prior to initiation, need for a support group, and value of home nursing.

**Conclusions:**

Caregivers of children on chronic dialysis had significantly poorer self-rated health and wellbeing compared with the general adult population. This may be due in part to their feelings of social isolation. Our findings highlight opportunities to improve caregivers' lived experiences.

## Introduction

Advanced Chronic Kidney Disease Stage 5 (CKD Stage 5D) has a significant impact on morbidity and mortality in the pediatric population, with a mortality rate reported up to 11% (1, 2). Dialysis is mostly utilized as a bridge to kidney transplant for children. It is estimated that 50–70% of children with CKD Stage 5D on dialysis receive peritoneal dialysis (PD) (3). PD is a home-based modality which allows children to participate in school and other extra-curricular activities more regularly, thereby providing opportunities for age-appropriate social interactions (1). While considered better for children's quality of life, PD places primary responsibility on caregivers who perform nightly dialysis as well as administer medications and often supplemental gastrostomy feeds (1). In the pediatric setting, in-center hemodialysis (HD) often occurs in outpatient hospital units performed by experienced medical staff, but the responsibility of administering medications, feedings, transportation to the dialysis unit and admissions for dialysis-related complications falls on caretakers.

A growing body of literature demonstrates the toll caring for children on chronic dialysis can take on caregivers. Tsai et al. reported on the significant psychosocial impact of caring for a child on peritoneal dialysis, highlighting the long-term impact (4). Tong et al. identified three major themes related to the experiences of parents of children with chronic kidney disease (CKD) (5), including intrapersonal issues (fatigue, stress, and uncertainty), interpersonal issues (medicalization of the parental role, disrupted peer relationships, and conflicts with medical teams), and external issues (pursuit of information, accommodation/finances, and balancing medical care with household responsibilities) (4). A recent study of caregiver experiences in pediatric dialysis in the United States identified four main themes through semistructured interview: caregiver medicalization, emotional adjustment, pragmatic adaptation and social adjustment (6). Collectively these studies highlight the increased medical, financial, and psychosocial responsibilities of parents of children with CKD Stage 5D and the shared experiences across several nations. A recent review noted that most of this literature focused on qualitative findings of caregiver experience in chronic pediatric dialysis (6). Few quantitative tools are currently available to assess caregiver experience (7–11).

Patient-reported outcomes (PROs) provide quantitative assessment tools of how a respondent feels, what they believe they are capable of doing, activities they can participate in, and evaluations of their health and wellbeing (12, 13). The growing importance of PROs in clinical research is highlighted by creation of the Patient-Centered Outcomes Research Institute and the increasing use of PROs in clinical trials and to support medical product claims approved by the American Food and Drug Administration (14). In 2004, the Patient Reported Outcomes Measurement Information System (PROMIS) was launched by the National Institutes of Health to advance the science of PROs. PROMIS's goal was to develop and evaluate the psychometric and clinical properties of self-reported measures of health, ensuring that they can be efficiently administered, offer precise assessments of health across a wide range of health status levels, are valid, and will be useful to clinicians and clinical researchers (15–17). Yet there are a limited number of studies evaluating the validity of PROMIS measures in caregivers of patients with chronic disease. Our study aimed to assess quality of life through PROMIS measures of the chronic pediatric dialysis caregiver population and provide additional qualitative information about their experience to improve family-centered care for children receiving chronic dialysis.

## Materials and Methods

### Data Source and Study Population

Participants were recruited from the Children's Hospital of Philadelphia Dialysis Program (in-center HD and home-based PD) during May 2018-June 2019. The study was approved by the CHOP Institutional Review Board. Caregivers were identified through review of patient medical records and clinic appointment schedules. Participation in the study was voluntary and required informed consent. All English-speaking caregivers of children on chronic dialysis were eligible. Caregivers were defined as a parent or guardian of a patient <18 years old who had received dialysis for ≧3 months. Non-English-speaking caregivers, those who cared for a child >18 years of age, caregivers of patients on dialysis for <3 months, and those who had a combined contact (phone and/or clinic approach) of more than 4 attempts were excluded. During the study period, 29 patients received chronic dialysis in our dialysis unit (14 PD and 15 HD).

### Patient Reported Outcomes

PROMIS tools previously used in an adult population were used in this study, including Fatigue Short Form 8a version 1.0 (18), modified Sleep Disturbance Short Form 8a version 1.0 (19) Positive Affect Short 15a version 1.0, Meaning and Purpose Short Form 8a version 1.0, and General Life Satisfaction Short Form version 1.0 (17). Data were collected on paper forms and input by study staff into a REDCap database (20, 21).

Participants also completed questionnaires which included caregiver demographics (age, gender, race, ethnicity, and relationship to child), annual family income level, hours per day spent on dialysis care (1–3, 4–6.9, 7–9.9, or 10–12.9 h) and free text questions pertaining to the experience of caring for a child on dialysis.

### Statistical Analysis

#### Quantitative Data Analysis

De-identified data were uploaded into the Health Measures Scoring Service^1^. Default calibration settings were selected for all tools. T-scores were based on a mean of 50 and standard error of 10 in the general population. T scores and demographic data were analyzed using STATA Version 14.2 software. Basic descriptive statistics (e.g., median and interquartile range for continuous variables and percentages for categorical variables) were used to describe the study sample.

#### Qualitative Data Analysis

Upon completion of the survey, we used thematic analysis. All free text written survey responses were independently analyzed by two members of our team (DLE and MRM) to inductively derive and identify concepts and themes related to pediatric dialysis caregiver experiences. Following these independent reviews, all responses were refined through iterative collaboration to further distill themes and subthemes until consensus was achieved. Saturation was achieved given repetition of caregiver-reported themes after 10 caregivers.

## Results

### Study Population

Twenty-five caregivers were enrolled in the study, including 18 caregivers of 11 children on PD and 7 caregivers of 6 children on HD (Table 1). Among all caregivers, 17 (68%) were female with a median age of 40 years. Fifteen (60%) identified as White and 9 (36%) identified as Black or African American. All caregivers identified as a parent of the child receiving dialysis. Seventeen children were included in this study. Eleven of whom were receiving peritoneal dialysis (mean age 8 years, range 2–17). Six patients received hemodialysis (mean age 12 years, range 3–17). The median age at initiation of dialysis was 3 years (range 0–16); 7 (41%) were female. Among PD caregivers, 3 (16.7%) reported they were the sole PD caregiver and 15 (83.3%) reported they shared PD responsibility. Of caregivers who shared responsibility for dialysis care, 58.6% ± 23 care was reported to be shared. Most PD caregivers (88.9%) reported spending more than 7 h per day on PD-related care; half of all caregivers specified this care required 10–12.9 h daily devoted to PD-related care.

**Table 1:** Pediatric dialysis caregiver and patient demographics.

	**Caregiver characteristics**		
	**Combined cohort**	**Caregivers PD**	**Caregivers HD**
	***n* = 25**	***n* = 18**	***n* = 7**
Age Yrs. (median, range)	40 (26–52)	39 (26–52)	41.5 (30–52)
Female gender *N* (%)	17 (68.0%)	12 (66.7%)	5 (71.4%)
Race			
White	15 (60.0%)	11 (61.1%)	4 (57.1%)
Black/African American	9 (36.0%)	7 (38.9%)	2 (28.6%)
Asian	1 (4.0%)	0 (0.0%)	1 (14.2%)
Ethnicity			
Hispanic/Latinx	1 (4.0%)	0 (0.0%)	1 (14.2%)
Relationship to child			
Mother	17 (68.0%)	12 (66.7%)	5 (71.4%)
Father	8 (32.0%)	6 (33.3%)	2 (28.6%)
**Peritoneal dialysis caregivers only**			
Hours (per day) spent on peritoneal dialysis-related care			
1–3 h	—	2 (11.1%)	—
4–6.9 h	—	0 (0%)	—
7–9.9 h	—	7 (38.9%)	—
10–12.9 h	—	9 (50.0%)	—
Sole peritoneal dialysis provider			
Yes	—	3 (16.7%)	—
No	—	15 (83.3%)	—
Percentage of PD Care Responsibility Reported by Provider		Mean 58.6% ± 23	—

Socioeconomic data from caregivers (Table 2) found that more than half (56%) of caregivers' annual family income was less than $50,000 (range less than $15,000–greater than $150,000). A minority (8%) reported challenges finding transportation. Nearly one-third (32%) of caregivers reported food insecurity in the home, and 16% reported difficulty meeting their child's dietary requirements.

**Table 2:** Self-reported socioeconomic characteristics of pediatric dialysis caregivers.

Annual family income	
Under $25,000	8 (32%)
$25,000–$49,999	6 (24%)
$50,000–$99,999	3 (12%)
Above $100,000	8 (32%)
Challenges finding transportation	
Yes	2 (8.0%)
No	22 (88.0%)
Not Answered	1 (4.0%)
Food Insecurity “Sometime in the last 12 months, the food I/we bought just didn't last and I/we didn't have money to get more.”	
Often	2 (8.0%)
Sometimes	6 (24.0%)
Never	17 (68.0%)
Difficulty meeting dialysis patient's recommended dietary requirements	
Often	3 (12.0%)
Sometimes	1 (4.0%)
Never	20 (80.0%)
Prefer not to answer	1 (4.0%)

### Caregiver Reported Outcomes–PROMIS Measures

As shown in Table 3, among all caregivers, positive affect (43.4 ± 10, [39.5–47.3]) and general life satisfaction (45.1 ± 11.5, [40.6–49.6]) were noted to differ significantly from the general population based on PROMIS average adjusted by Health Measures Scoring Service T-score, standard deviation, and 95% confidence interval. In subanalysis of peritoneal dialysis caregivers, scores for measures of fatigue (52.8 ± 8.5, [51.9–53.7]) and sleep disturbance (51.4 ± 11.8, [50.1–52.7]) were higher, while scores for positive affect (43.8 ± 8.5, [39.9–47.7]) and general life satisfaction (44.8 ± 9.7, [40.3–49.3]) were lower compared to the general population. Only the meaning and purpose measure for PD caregivers was on par with the general adult population. In the HD caregiver population, sleep disturbance (58.6 ± 7.9, [52.8–64.5]), positive affect [42.6 ± 5.3, (38.7–46.5)], and meaning and purpose [57.0 ± 9.2, (50.2–63.8)] were noted to be different than the general adult population.

**Table 3:** PROMIS caregiver average adjusted T-score, confidence interval, and SD.

**Promis measure**	**Combined cohort (*N* = 25)**	**Peritoneal dialysis (PD) (*N* = 18)**	**Hemodialysis (HD) (*N* = 7)**
	**Avg. adjusted T-score, SD (95% CI)^**a**^**	**Avg. adjusted T-score, SD (95% CI)**	**Avg. adjusted T-score, SD (95% CI)**
Fatigue	53.2 ± 10 (49.3–57.1)	52.8 ± 8.5 **(51.9–53.7)**	54.0 ± 5.5 (49.9–58.1)
Sleep disturbance	53.4 ± 14 (47.9–58.9)	51.4 ± 11.8 **(50.1–52.7)**	58.6 ± 7.9 **(52.8–64.5)**
Positive affect	43.4 ± 10 **(39.5–47.3)**	43.8 ± 8.5 **(39.9–47.7)**	42.6 ± 5.3 **(38.7–46.5)**
General life satisfaction	45.1 ± 11.5 **(40.6–49.6)**	44.8 ± 9.7 **(40.3–49.3)**	45.9 ± 6.6 (41.0–50.8)
Meaning and purpose	53.4 ± 16.5 (46.9–59.9)	52.0 ± 15.2 (45.0–59.0)	57.0 ± 9.2 **(50.2–63.8)**

a *T-scores based on mean of 50 and standard error of 10 in the general reference population.*

*Highlighted results, where 95% CI excludes the mean of 50, indicate a statistically significant differences as compared to the reference population*.

### Caregiver Experiences

Self-reported caregiver experience focused around themes of financial hardship, loss of career, relationship strain, loss of social connections, impact on travel, increased anxiety, strain on future planning, sleep interference, and helplessness. The most reported themes were: impact on travel, increased anxiety and overwhelming nature of dialysis.

Some caregivers reported gaining emotional support from other dialysis caregivers and finding online parent support groups. One caregiver specified the burden of care was decreased when their child transitioned from home PD to in-center HD.

When asked how the medical team could improve caregiver experience, caregivers felt the team could facilitate more opportunities for peer support, educate about specialized clothing for dialysis patients, discuss duration of dialysis therapies prior to initiation, and facilitate smoother transitions between segments of nephrology care (e.g., CKD to dialysis, expectations of time on dialysis prior to transplant). Caregivers also expressed the need for more guidance on potential complications of dialysis (e.g., education on side effects of treatment and infectious complications), time needed to learn how to perform dialysis, anticipated costs of dialysis, increased space needed for in-home PD supplies, and financial assistances (e.g., parking passes, meal vouchers, transportation assistance).

Caregivers also expressed the value of in-home assistance (e.g., home health aides and nursing), which allow parents time for respite/self-care and to provide basic needs for themselves, their child on dialysis, and other family members. One caregiver noted home nursing focused on care of their child on dialysis created time to cook meals recommended for the child's restricted diet. Another caregiver noted, “having nursing help [for peritoneal dialysis] should be mandatory in the beginning. All PD parents need it.” It should be noted that nursing services were made available for adjunct assistance in caring for PD patients (administering medications and feeds, assisting with vital signs), but never performing the actual dialysis procedure.

### Qualitative Analysis

Through iterative analysis of free text responses provided by caregivers, four themes of pediatric dialysis caregiver experience were identified; three of the four central themes were further divided into subthemes as follows: (1) loss (social loss and loss of normalcy), (2) psychological strain (helplessness, anxiety, and overwhelming nature of dialysis), (3) financial hardship, (4) resiliency/coping mechanisms (peer support, opportunities for respite), see Table 4.

**Table 4:** Themes and subthemes of pediatric caregiver reported experience.

**Loss**	
*Social connection*	“We became lost as a couple as focus turns to being medical providers and caregivers. Losing identity as a couple and not being able to go out as a couple.” “We felt ‘stuck' in the bedroom a lot and was somewhat depressing hearing kids playing and seeing neighbors talking and we were always upstairs.” “[W]e no longer go out really.“ “Tough keeping outside friendships with care needed.”
*Normalcy*	“[Dialysis] has completely changed our lives. We are confined to be by his machine 12 h/daily” ”[Dialysis] [r]estricted [our lives]. No camping, no sleepovers, etc. Food Restrictions.“ “Need to sleep in same room as him [patient], and awake a few times a night (e.g., machine beeps, cord tangling...)” “It's certainly life changing but you learn to make the best of situations and not letting it consume you.”
**Psychological strain**	
*Helplessness*	“I honestly wouldn't know where to start. The main thing is being a mom, and not being able to take your child's pain away.” “... worrying how long he will be on PD and his waiting long to get transplant, his future life.”
*Anxiety*	“I have anxiety about leaving the house with him at times.” “I was scared to open up that it would make me look vulnerable or depressed when I was trying to work through dealing with my new life for a while.”
*Overwhelming nature of performing dialysis*	“Everyone talks at once sometimes. It's a lot of info + overwhelming. ” “I wish I was directed from the beginning to begin therapy and prepare my mental state of the shock factor and stress of being 100% responsible to keep a child alive on a machine.”
**Financial hardship**	
	“[Life is] difficult and changed completely: loss of career, financially hard. Ninty percentage of focus on care of child.” “Fear of something going wrong, work, too much time taken off, fear of losing my job.”
**Resiliency/coping mechanisms**	
*Peer support*	“Amazing listening and helping when I opened up....Being introduced to other families helped me a lot to not feel alone! Thank you for this. Helping to find more support online would have been more helpful though. Help deciding best place to do it based upon amount of hours.”
*Opportunities for respite*	“[PD gave] [m]ore time at home compared to HD” “[PD] is better than HD-we are home more” “Having nursing help should be mandatory in the beginning. I wish I knew how life changing it would be... I wish I hadn't denied home nursing. all PD parents need it.” “Assist parents on getting home aids or help at home so we can cook proper meals, rest properly. This will help parents concentrate on better nutrition and care for the child. Also, assisting on free transportation, meal vouchers, parking passes, will ease the stress we all go through when coming for so many times to a hospital.”

Additionally, caregivers were asked how the medical team could improve caregiver experience. Increased support in 4 major areas was requested, including: (1) peer support opportunities, (2) smoother transitions in care from CKD management to dialysis, (3) areas of increased guidance, and (4) opportunities for more tangible supports. Comments provided by caregivers included need for mental health support: “I wish I was directed from the beginning to begin therapy and prepare my mental state of the shock factor and stress of being 100% responsible to keep a child alive on a machine” as well as nursing support opportunities: “Having nursing help should be mandatory in the beginning.”

## Discussion

Patient Reported Outcomes have grown in recent years, largely because they provide primary insight into how disease, illness, and treatments affect patients and their caregivers. In this study, PRO tools were used to quantify the burden of being a caregiver of a child on chronic dialysis. Previous studies have demonstrated the physical, psychological, social, and financial burdens experienced by caregivers of children on dialysis from a qualitative perspective (5, 6, 22–28). To our knowledge, this study is the first to use PROMIS measures to evaluate caregiver experience in the pediatric dialysis population and is one of the first to use these tools in caregivers of any pediatric patient with chronic illness (29, 30). Use of PROMIS measures in caregivers of children on chronic dialysis sheds light on targets for future interventions to improve both caregiver and patient quality of life.

The PROMIS measures used in our study were developed for the general adult population. These measures are appropriate for assessment of pediatric dialysis caregivers since they are representative of a general adult population. In accordance with validated PROMIS analysis, we used an overall approach of considering one standard deviation above or below the mean as meaningful. This may underpower our results in a relatively small population (though this is often the case with pediatric dialysis) [(31, 32), see text footnote 1, respectively]. Despite this sample size and reduced power, our data highlight the impact that being a pediatric dialysis caregiver can have on positive affect and general life satisfaction. Similar to findings in other studies, caregivers in our cohort were more likely to be female (mothers) and experience additional challenges such as poverty and food insecurity that negatively impact quality of life [(6, 26, 33, 34), see text footnote 1, respectively].

In the pediatric HD caregiver cohort, a significant impact on sleep disturbance, positive affect, and meaning and purpose was noted. The impact on sleep disturbance was higher in this population than in the PD caregiver cohort, which was unexpected. This may be due to overall poor sleep in the pediatric CKD population, but given the limited number of HD caregivers in our pilot study (7 caregivers), these findings should be investigated in a larger cohort (35). Qualitative methods, such as focus groups or interviews, could elucidate factors, if any, that affect HD caregivers. HD caregivers also reported an above the mean score in meaning and purpose; attention should paid to this scoring in larger studies to see if this finding remains significant.

In the pediatric PD caregiver cohort, a significant impact on fatigue, sleep disturbance, positive affect, and general life satisfaction was noted. As a primarily nocturnal-based therapy, the findings of significant fatigue and sleep disturbance are expected. Most caregivers (83.3%) reported sharing PD-related care approximately equally with another caregiver (mean 58.6% ± 23), while three (16.7%) were the sole caregiver. Half of PD caregivers reported spending 10–12.9 h per day on PD-related care for their child. This time included administering medications multiple times per day, a heavily restricted diet or tube feeding regimen, and twice daily vital signs (often with manual blood pressure measurements) in addition to performing home-based PD. These responsibilities, even when shared with another caregiver, are time-consuming, demanding, and lead to diminished quality of life, especially without identifiable sources of respite.

Themes distilled from provided qualitative data add to the richness of the PRO data by highlighting specific themes and potential opportunities for interventions provided by the caregivers in their own words. Our qualitative analysis echoed those of previous groups highlighting themes of loss, psychological strain, financial hardship, and resiliency/coping mechanisms. Improvement strategies focused on increased support opportunities, particularly during dialysis initiation and periods of transition. To allow for different learning styles, dialysis education should be offered in visual, verbal, and hardcopy formats to allow caregivers resources to reference later. Teams should also be cognizant of financial barriers and burnout. We suggest regular psychosocial screening to identify impending caregiver burden, need for additional support, and time to make additional interventions.

CKD Stage 5D requiring chronic dialysis is a rare outcome in children. Although our cohort is small, it is sizeable for a single pediatric dialysis center and represents a more diverse population than other published works (29). Further research into the patient and caregiver experience on dialysis should be explored through a multicenter study design to adequately power these results. At the pilot stage, we collected limited socioeconomic data and did not collect geographic dispersement or travel time data, factors which could impact caregiver burden and the decision of which dialysis modality a patient receives. These are limitations we acknowledge and hope to remedy with the next stage of work. Due to limitations in funding we excluded caregivers without English proficiency. We acknowledge this may introduce selection bias into this work and plan to add budget requirements for interpreter and translation services to future projects. Nonetheless, our results are thought-provoking and highlight the need to expand this pilot study to a broader population through multicenter collaboration.

In a recent review of caregiver burden amongst pediatric dialysis caregivers, Wightman highlights many qualitative studies demonstrating caregiver burden in pediatric dialysis internationally as offering “the possibility to better understand broader psychosocial impacts . . . [ideally], themes and concepts described in qualitative research should be affirmed by quantitative studies” (6). Use of PROMIS measures in the clinical setting provides quantitative tools to identify caregivers who are struggling with their children's care and valuable data on the degree of individual caregiver burden over time. Measuring this burden over time and its impact on caregiver wellbeing can help pediatric nephrologists and the multidisciplinary dialysis care team to identify those caregivers at greatest need for more resources, support, and respite.

## Data Availability Statement

The raw data supporting the conclusions of this article will be made available by the authors, without undue reservation.

## Ethics Statement

The studies involving human participants were reviewed and approved by Children's Hospital of Philadelphia IRB. Written informed consent to participate in this study was provided by the participants' legal guardian/next of kin.

## Author Contributions

MM, DL, MD, SF, CF, and SR: study conception and design. DL, MM, MT, and SR: acquisition of data. MM, DL, MD, CF, SF, MT, and SR: analysis and interpretation of data. All authors were involved in drafting the article or revising it critically for important intellectual content and approved the final version submitted.

## Funding

This study is part of the NIH PEPR Consortium, a multi-center collaborative studying how patient-reported outcomes change over time in response to pediatric chronic illness and its treatments. Funding is from the NIH, The Child-Centered Outcomes in Practice and Research (COPR) Center of Excellence: Strengthening the Clinical Validity Evidence Base for PROMIS Measures in Chronically Ill Children grant U19AR069525 (PI: CF). DL was funded by T32-DK007006-43. MM was funded by T32-DK007785.

## Conflict of Interest

The authors declare that the research was conducted in the absence of any commercial or financial relationships that could be construed as a potential conflict of interest.

## Publisher's Note

All claims expressed in this article are solely those of the authors and do not necessarily represent those of their affiliated organizations, or those of the publisher, the editors and the reviewers. Any product that may be evaluated in this article, or claim that may be made by its manufacturer, is not guaranteed or endorsed by the publisher.

## Footnotes

^1^ https://www.healthmeasures.net/
